# Editorial: Developing strategies to improve diabetes management in college-going young adults

**DOI:** 10.3389/fendo.2024.1402133

**Published:** 2024-04-10

**Authors:** Mridusmita Saikia, Zohra S. Lassi, Anthony L. McCall

**Affiliations:** ^1^ Meinig School of Biomedical Engineering, Cornell University, Ithaca, NY, United States; ^2^ Robinson Research Institute, University of Adelaide, Adelaide, SA, Australia; ^3^ University of Virginia School of Medicine, Charlottesville, VA, United States

**Keywords:** type 1 diabetes mellitus (T1DM), glucose sensing, community engagement, diabetes workshop, diet, lifestyle

## Introduction

Navigating the transition from life at home to university can be often challenging, more so for the new generation of students entering university after a COVID-ravaged high school period. This change is even more difficult for students with Type 1 diabetes mellitus (T1DM), who must navigate this social and physical transition while assuming increasing responsibility for their diabetes care. These pressures may sometimes lead to disengagement from medical care (1). The number of young adults affected by T1DM is increasing globally. Studies have even suggested that SARS-CoV-2 may be the underlying cause of new-onset T1DM (2–4). Thus, there is an urgent need for research into T1DM, particularly focusing on creating treatment options, clinical services, and social support for college-going patients. This Research Topic aims to bridge some of the gaps in the current understanding of T1DM in young adults and provide a roadmap for creating more youth-friendly treatment and support options.

## Themes highlighted in the Research Topic

### Creating treatment options for college students with T1DM

T1DM is a condition in which the body can no longer produce sufficient amounts of its own insulin (5). The most common treatment for a person with T1DM is insulin supplementation (6). This is administered through injections at set times of the day and mealtimes (prandial). For optimal management, people with T1DM need to be aware of the ratio of their prandial insulin dose to carbohydrate intake, premeal blood glucose levels, and anticipated physical activity. They should also be capable of measuring their blood glucose levels and managing insulin dosing under various circumstances. All of this can become overwhelming for a person juggling academic and social pressures.

Alternatively, a person with T1DM may use an insulin pump. Currently, the most advanced commercially available technology for glucose monitoring and automatic delivery is a single-hormone (SH [insulin]) closed-loop system, also known as an artificial pancreas (AP). Even this advanced technology, might induce periods of hypoglycemia, especially, during intense physical activity. A study in this Research Topic by Lindkvist et al. tested whether their new dual-hormone (DH [insulin and glucagon]) closed-loop system can provide better glycemic control. Their results indicate that the DH system is not as efficient as the SH system. Another study in this Research Topic, conducted by Liu et al. has created an intelligent controller that may be combined with AP technology for better control of blood glucose levels. In-silico tests show that the controller is effective in preventing hyperglycemia, however, it does not attenuate the risk of hypoglycemia.

An uncomplicated medication routine might alleviate pressure on college students with T1DM. In this Research Topic, Lagunas-Rangel et al. report that a triple-drug combination therapy consisting of gamma-aminobutyric acid (GABA) together with a dipeptidyl-peptidase-4 inhibitor (DPP-4i), and a proton pump inhibitor (PPI), has a superior therapeutic effect in improving diabetes parameters in NOD mice, an animal model for human T1DM. The authors comment that their inexpensive triple-drug regimen is an accessible treatment option for T1DM. Another article in this Research Topic by Almutair and Almulhem reports that treatment with Semaglutide, a GLP-1RA, administered weekly over 4 months, led to a notable improvement in an 18-year-old, non-obese female diagnosed with Hepatocyte nuclear factor-1B maturity-onset diabetes of the young (MODY).

### T1DM management through diet and weight management

In addition to medication, maintaining a healthy lifestyle can play a crucial role in the management of T1DM. The study by Li et al. shows that high sugar intake in the diet can be linked to the development of T1DM in NOD mice. Obesity is another factor often associated with diabetes. Sheng et al. monitored the body mass index (BMI), waist circumference (WC), and waist-height ratio (WHtR) of 12,823 normoglycemic individuals to begin with, for 12 years. They report that WC is highly correlated with the occurrence of diabetes in the short term (2-5 years), while WHtR is correlated with the occurrence of diabetes in the long term (6-12 years).

Maintaining a healthy lifestyle can be tricky in college; the tendency for quick meals and late-night snacking can lead to hyperglycemia and obesity. Explaining the implications of such behavior can be key to preparing young adults with T1DM for college life.

### T1DM management through education, social support and sustained monitoring

A study in this Research Topic by Wolf et al. provides an account of the workshop they created and implemented to prepare young adults with T1DM and their parents, for life in college. In addition to valuable insights into academic life, the workshop provided opportunities to interact with endocrinologists and nutritionists. One key piece of advice was to set up a diabetes care provider in their new place of residence as soon as possible.

Monitoring health parameters is key to managing T1DM. Catamo et al. in their study involving 324 subjects with T1DM aged <21 years, show that markers of diabetic complications, such as dyslipidemia, can occur in individuals as young as 11. Long waiting times for specialist appointments might aggravate such conditions, leading to complications. Dupenloup et al. discuss an interesting alternative. A new, telemedicine-based care model based on the use of a remote patient monitoring tool that analyzes continuous glucose monitoring data and identifies individuals likely to benefit from contact with the diabetes care team. Dupenloup et al. evaluated the financial impact of adopting this tool by pediatric endocrinology clinics and reported that it is financially sustainable with insurance. This can be a medical intervention tool that students rely on while waiting to obtain specialist appointments.

Mental health is another important concern for people with T1DM as well as their family members. In their review, Chen et al. discuss the high prevalence of depression among parents of children/adolescents with T1DM. The authors state that higher instances of social discrimination, marginalization and stigma experienced by these patients and their parents might contribute to this observation. A workshop such as the one created by Wolf et al. can provide opportunities for interaction and social support for people with T1DM and their parents.

## Summary and future directions

Easy and reliable treatment options will go a long way in providing support to students with T1DM. However, we are still far from effective insulin alternatives. In this Research Topic, Liu et al. discuss the use of traditional Chinese medicine to regulate the microbiome of people with diabetes as a treatment option. Immunotherapy has been another avenue of research (7, 8). While we await a breakthrough, this Research Topic highlights the importance of preparation through education and counseling, access to health monitoring, and support for a healthy lifestyle in the smooth transition of young adults with T1DM from home to their place of higher education.

## Author contributions

MS: Conceptualization, Writing – original draft, Writing – review & editing. ZL: Writing – review & editing. AM: Writing – review & editing.
